# Should analyses of large, national palliative care data sets with patient reported outcomes (PROs) be restricted to services with high patient participation? A register-based study

**DOI:** 10.1186/s12904-020-00596-z

**Published:** 2020-06-23

**Authors:** Maiken Bang Hansen, Morten Aagaard Petersen, Lone Ross, Mogens Groenvold

**Affiliations:** 1 Department of Palliative Medicine, Bispebjerg and Frederiksberg Hospital, DK-2400 Copenhagen, Denmark; 2 Department of Public Health, University of Copenhagen, DK-1014 Copenhagen, Denmark

**Keywords:** ‘Palliative care’, ‘Quality of Life’, ‘Sign and Symptoms’, ‘Functioning’, ‘Patient Reported Outcome Measures’, ‘Needs assessment’, ‘Symptom assessment’, ‘Patient participation’, ‘Response rate’, ‘Selection Bias’

## Abstract

**Background:**

There is an increased interest in the analysis of large, national palliative care data sets including patient reported outcomes (PROs). No study has investigated if it was best to include or exclude data from services with low response rates in order to obtain the patient reported outcomes most representative of the national palliative care population. Thus, the aim of this study was to investigate whether services with low response rates should be excluded from analyses to prevent effects of possible selection bias.

**Methods:**

Data from the Danish Palliative Care Database from 24,589 specialized palliative care admittances of cancer patients was included. Patients reported ten aspects of quality of life using the EORTC QLQ-C15-PAL-questionnaire. Multiple linear regression was performed to test if response rate was associated with the ten aspects of quality of life.

**Results:**

The score of six quality of life aspects were significantly associated with response rate. However, in only two cases patients from specialized palliative care services with lower response rates (< 20.0%, 20.0–29.9%, 30.0–39.9%, 40.0–49.9% or 50.0–59.9) were feeling better than patients from services with high response rates (≥60%) and in both cases it was less than 2 points on a 0–100 scale.

**Conclusions:**

The study hypothesis, that patients from specialized palliative care services with lower response rates were reporting better quality of life than those from specialized palliative care services with high response rates, was not supported. This suggests that there is no reason to exclude data from specialized palliative care services with low response rates.

## Background

It has been shown that not all symptoms in advanced cancer patients admitted to palliative care are recognized by the health care professionals. Systematic symptom assessment has been proposed as a solution to this problem [1–3]. Therefore, starting in 2010, cancer patients in all specialized palliative care (SPC) services in Denmark have been invited to complete the European Organisation for Research and Treatment of Cancer Quality of Life Questionnaire-Core-15-Palliative Care questionnaire (EORTC QLQ-C15-PAL) at the start of SPC. The questionnaire assesses different quality of life aspects of cancer patients in palliative care, i.e., nine symptoms and problems and overall QOL. There is increasing interest in the analysis of large, national data sets from palliative care with patient reported outcomes (PROs). Thus, we planned to analyze these patient reported QOL data to get a better understanding of cancer patients’ QOL at the start of SPC on a national level. However, the response rates varied to a large extent between the SPC services in Denmark. This led to a dilemma: we were concerned that the data from palliative services with low response rate might be affected by selection bias (e.g., if fewer of the most symptomatic patients were included, the resulting scores would not be representative). On the other hand, excluding some of the SPC services from the dataset, would lead to reduced generalizability of our study findings: we would not be reporting national data but data from ‘well-performing services’ only. And if excluding services, where should the cutoff for exclusion be?

Some studies on patients with advanced cancer and in palliative care have found that health affected study participation [4, 5]: lower performance score and more pain were associated with a lower probability of answering a questionnaire [4] and non-respondents had lower physical performance and shorter survival than respondents [5]. However, a third study in patients with advanced cancer found no indication of clinically relevant differences in the quality of life scores when observed scores were compared to scores that included imputed data, and thus found no indication of bias due to non-participation [6]. Thus, non-respondents would likely either be similar or in worse health compared to respondents.

Since patients admitted to SPC are severely ill and close to death, it is not feasible for all patients to report their symptoms/problems. However, if research is to give an understanding of different aspects of QOL (symptoms, problems and overall QOL, in the following generally referred to as QOL) at the start of SPC it is crucial to obtain QOL reporting from as representative a sample as possible. If better QOL-scores are reported in SPC services with low response rates it could be because only the most well patients (i.e. those who were easiest to obtain QOL reporting from) were asked to report their QOL. In this case, the QOL scores from SPC services with low response rates would probably be biased and thus overestimate QOL, and one could argue that a more correct estimate of QOL could be obtained if services with low response rates were excluded from future analyses.

Therefore, in this study, we wished to test the hypothesis: *Patients from SPC services with low response rates report (on average) better on different QOL aspects than patients from services with high response rates, indicating a (larger) impact of selection bias in services with low response rates than in services with high response rates*. Thus, although it is impossible to rule out selection bias even in services with quite high response rates, we assumed that differences in average scale scores between services with low and high response rates could reflect possible selection bias in the services with low response.

Thus, the aim of this study was to test whether response rate was associated with the level of different QOL aspects (i.e., with the level of the nine symptoms and problems and overall QOL from the EORTC QLQ-C15-PAL questionnaire).

If our study hypothesis was rejected and no indication of bias was found, there would not seem to be any reason to exclude data from the SPC services with low response rates in future studies.

## Methods

### Patients and data

#### The Danish palliative database

All SPC services in Denmark deliver data to the Danish Palliative Database (DPD) on patients referred to their service. DPD contains information on all patients referred to SPC in Denmark from 2010 and onwards. Patient information recorded in DPD includes diagnosis, socio-demographic factors, whether the patient has received SPC, and the symptom/problems and QOL among patients admitted to SPC. Data in this study was obtained from the DPD.

#### Inclusion criteria

This study included data for patients who: 1) were admitted to SPC and died between January 1st, 2010 and December 31st, 2015, 2) had a cancer diagnosis, 3) were 18+ years of age and 4) answered the EORTC QLQ-C15-PAL questionnaire in the period from 3 days prior to admission to the day of admission to SPC.

#### EORTC QLQ-C15-pal

The EORTC QLQ-C15-PAL questionnaire is a shortened version of the widely used EORTC QLQ-C30 developed for assessment of different aspects of quality of life in cancer patients in palliative care, i.e., assessment of nine symptoms and problems and overall QOL [7]. EORTC QLQ-C15-PAL has four multi-item scales (physical functioning, emotional functioning, fatigue and pain) and six single-item scales (dyspnea, insomnia, appetite loss, constipation, nausea/vomiting and overall QOL) [7]. The patients answer on a 4-point scale how much they have experienced the symptom/problem (not at all, a little, quite a bit, very much), except for overall QOL which is rated on a 7-point scale where 1 is very poor and 7 excellent. The time frame is the past week except for physical functioning for which no time-period is specified.

### Statistics

The analyses were performed using SAS statistical software version 9.4.

#### Conversion of scale scores and computation of response rate

The responses to the EORTC QLQ-C15-PAL were converted into 0–100 scales according to the scoring manual [8, 9]. For the two functional scales and QOL, higher scores represent better functioning/QOL, whereas for the seven symptom scales, higher scores represent worse symptoms [8, 9].

The response rate was computed for each SPC service for each calendar year. The response rate for a service a given year was computed as the number of patients admitted to the service that year who completed the questionnaire at admittance divided by all the patients who were admitted to the service the same year. A response rate was allocated to each patient in the study. Thus, a patient admitted to e.g., the Palliative Care Team in Århus in 2010 was allocated the response rate of Palliative Care Team in Århus in 2010. Response rate was grouped into; < 20.0%, 20.0–29.9%, 30.0–39.9%, 40.0–49.9%, 50.0–59.9% and ≥ 60.0%.

#### Multiple linear regression

Multiple linear regression analyses were performed to study the association between response rate and scale scores. The ≥60.0% response rate group was used as reference since it was expected to have the least selection bias.

The study hypothesis, i.e. that patients from SPC services with low response rates had better scale scores on the ten QOL aspects than patients from services with high response rates (indicating possible selection bias), was tested using four criteria:

1. The *p*-value for the *overall* association between response rate and scale score was < 0.05.
2. The p-value for at least one pairwise comparison of scale scores in lower response rates groups (< 20.0%, 20.0–29.9%, 30.0–39.9% 40.0–49.9%, 50.0–59.9%) with the ≥60.0% response rate group was < 0.05.
3. The difference in scale score was in the direction supporting our hypothesis (lower symptom score and higher functioning and QOL in the lower response rate groups compared to the ≥60.0% group), and
4. The mean difference in scale scores had to be 5 or more to be considered clinically relevant.

If one or more of the criteria were not fulfilled, the study hypothesis was rejected. This was tested for the scale scores of each of the ten QOL aspects.

The choice of 5 as a clinically relevant difference in scale scores was based on results and conclusions from previous studies [10–14]. In these studies, 10 is often used as the clinically relevant cut point [15]. In this study, the more conservative cut point of 5 was chosen because it was important not to miss relevant differences.

#### Adjustment and random effects in the regression analyses

The patients in this study were from either hospices or palliative care teams. Previous studies have found more symptoms, worse performance status and shorter survival time in hospice patients compared to patients from other palliative care services [16–19]. We therefore controlled for type of SPC service (hospice/team) in the regression analyses. A random effect for SPC service was also included in the model because patients from the same SPC service were expected to be more similar than patients from different SPC services. Further, a random effect of patient id was included in the model to account for the fact that some patients filled in more than one questionnaire (if admitted to more than one service).

#### Choice of regression analysis method

The results of the association between response rate and scale scores from the linear regression analyses (mean differences in symptom score) are presented in this article due to their simple interpretation. Linear regression is, however, not the obvious choice for the scales with only four (dyspnea, sleep, appetite loss, constipation and nausea) or seven (pain, emotional functioning and QOL) possible scores because the normal distribution assumption is likely to be violated. Logistic regression (with symptom scores dichotomized at the median) with random effects was therefore performed as a sensitivity analysis to ensure that significant associations were not missed in the linear regression analyses.

#### Change in mean scale scores when excluding patients from SPC services with response rates < 60.0%

In addition to the regression analyses we compared the mean scale scores for the entire study sample with the mean scores obtained when patients from services with response rate < 20.0, < 30.0, < 40.0, < 50.0 and < 60.0%, respectively, were excluded. Higher symptom/lower functional scores in the reduced samples would support the hypothesis of selection bias.

## Results

### Study population

The 40,316 adult Danish cancer patients in DPD who received specialized palliative care and died between 2010 and 2015 had 49,307 admittances to SPC services (some patients were admitted to more than one service). In 24,589 (49.9%) of these patient admittances, based on 22,420 patients, the EORTC QLQ-C15-PAL was answered. These 24,589 admittances were included in this study.

Of the 24,589 SPC admittances, 49.0% represented women and the average age was 68.5 years (Table 1). Three of four (74.0%) of the SPC admittances were in a palliative care team. The number of SPC admittances increased from 2010 to 2014 but decreased from 2014 to 2015 (patients who died later than 2015 were not included). The largest differences between patients responding to the EORTC QLQ-C15-PAL questionnaire (and thus included in the study) and non-respondents was that the study population were less likely to be hospice patients (26.0% vs. 47.9%) and their average survival time was longer (94.0 vs. 64.5 days) compared to non-respondents (Table 1).

**Table 1:** Characteristics of the study population (i.e., those who answered the EORTC QLQ-C15-PAL at the start of palliative care) and non-respondents

	Answered EORTC QLQ-C15-PAL			
	Yes		No	
	N	%	N	%
All	24,589	100	24,718	100
Age				
Mean	68.5		69.2	
Median	69		70	
Range	19–101		18–105	
Gender				
Women	12,150	49.4	12,695	51.4
Men	12,439	50.6	12,023	48.6
Cancer site/diagnosis				
Head and neck	765	3.1	730	3.0
Esophagus	844	3.4	738	3.0
Stomach	772	3.1	700	2.8
Small Intestine	177	0.7	160	0.7
Colon and rectum	2984	12.1	2795	11.3
Liver and intrahepatic bile ducts	828	3.4	887	3.6
Pancreas	1878	7.6	1735	7.0
Lung, bronchus and trachea	6381	26.0	6250	25.3
Melanoma	518	2.1	549	2.2
Breast	2003	8.2	1986	8.0
Cervix	248	1.0	246	1.0
Uterus	277	1.1	311	1.3
Ovary	886	3.6	870	3.5
Prostate	1843	7.5	1629	6.6
Bladder	617	2.5	710	2.9
Kidney, renal pelvis, ureter	753	3.1	700	2.8
Brain and central nervous system	637	2.6	1091	4.4
Lymphoma	158	0.6	225	0.9
Myelomatosis	205	0.8	210	0.9
Leukaemia	225	0.9	331	1.3
Sarcomas and other soft tissues	298	1.2	281	1.1
Other cancer site	767	3.1	831	3.4
Unknown cancer site	525	2.1	753	3.1
Specialized palliative care service				
Palliative care teams	18,207	74.0	12,880	52.1
Hospice	6382	26.0	11,838	47.9
Survival time from start of specialized palliative care to death (days)				
Mean	94.0		64.5	
Median	42		20	
Range	0–2126		0–2101	
Year of admission				
2010	2716	11.0	4172	16.9
2011	3400	13.8	4528	18.3
2012	4364	17.7	4164	16.8
2013	4929	20.0	4054	16.4
2014	5019	20.4	4076	16.5
2015	4161	16.9	3724	15.1
Response rate^a^				
< 20%	602	2.5	5501	22.3
20.0–29.9%	1282	5.2	3957	16.0
30.0–39.9%	2145	8.7	4183	16.9
40.0–49.9%	2926	11.9	3537	14.3
50.0–59.9%	3442	14.0	2795	11.3
≥ 60.0%	14,192	57.7	4745	19.2
Number of questionnaires completed per patient^b^				
1	20,285	90.5	–	–
2	2102	9.4	–	–
3	32	0.1	–	–
4	1	0.0	–	–
EORTC scale-scores (mean, range)				
Pain	56.4 (0.0–100.0)		–	–
Dyspnea	58.0 (0.0–100.0)		–	–
Sleeplessness	37.0 (0.0–100.0)		–	–
Appetite loss	58.3 (0.0–100.0)		–	–
Constipation	33.3 (0.0–100.0)		–	–
Fatigue	75.9 (0.0–100.0)		–	–
Nausea/vomiting	24.8 (0.0–100.0)		–	–
Emotional function	64.8 (0.0–100.0)		–	–
Physical function	27.4 (0.0–93.3)		–	–
Overall quality of life	39.0 (0.0–100.0)		–	–

^a^ Response rate was computed according to SPC service and calendar year. Thus, a patient admitted to an SPC service in 2012 was allocated the response rate of that SPC service for 2012. ^b^Only one questionnaire could be completed per SPC admittance and if patients were admitted more than once to the same SPC service, only the first was included

### Response rate

The patients were from 44 services, and each service admitted between 2 and 591 patients each year. The overall response rate was 49.9% and varied between year and service from 0.0 to 93.2% (Table 2).

**Table 2:** Number of patients admitted to SPC and response rate by admission year and overall (*N* = 49,307)

	Year of admittance to specialized palliative care (SPC)													
	**2010**		**2011**		**2012**		**2013**		**2014**		**2015**		**Total**	
**SPC service**	N	RR	N	RR	N	RR	N	RR	N	RR	N	RR	**N**	**RR**
Anker Fjord Hospice	115	26.1	159	46.5	163	46.0	167	78.4	161	86.3	173	52.0	**938**	**57.5**
Arresødal Hospice	175	0.0	186	0.0	161	3.7	209	15.8	204	20.1	170	18.8	**1105**	**10.1**
Diakonissestiftelsens Hospice	204	12.7	223	22.9	189	31.2	188	45.2	169	63.9	153	48.4	**1126**	**35.8**
Gudenå Hospice	NE	NE	NE	NE	NE	NE	NE	NE	NE	NE	74	20.3	**74**	**20.3**
Hospice Djursland	196	43.4	172	47.7	186	46.8	204	44.1	220	51.4	186	33.3	**1164**	**44.6**
Hospice Filadelfia	136	75.7	163	76.1	194	80.9	169	68.6	182	62.6	160	72.5	**1004**	**72.7**
Hospice Fyn	131	30.5	171	34.5	159	40.3	121	26.4	124	20.2	117	18.8	**823**	**29.4**
Hospice Limfjorden	146	38.4	141	34.0	173	32.4	190	38.4	173	54.9	171	56.7	**994**	**42.8**
Hospice Sjælland	10	0.0	186	0.0	183	33.9	201	18.9	266	15.8	243	13.2	**1089**	**16.0**
Hospice Sydfyn	NE	NE	NE	NE	24	33.3	137	30.7	173	37.0	127	55.1	**461**	**39.9**
Hospice Sydvestjylland	145	0.0	139	18.0	149	12.8	126	11.9	160	27.5	145	29.0	**864**	**16.8**
Hospice Søholm	114	0.9	134	2.2	149	8.7	142	24.6	144	47.9	122	38.5	**805**	**20.9**
Hospice Sønderjylland	107	56.1	130	51.5	149	58.4	135	25.9	122	27.0	112	43.8	**755**	**43.8**
Hospice Vendsyssel	76	0.0	89	0.0	89	0.0	122	0.8	119	12.6	134	9.7	**629**	**4.6**
Kamilianergaarden Hospice	141	2.1	190	7.9	172	11.6	179	30.2	179	43.0	154	47.4	**1015**	**23.8**
PCT Bispebjerg	378	31.7	401	30.2	345	32.5	363	25.6	341	44.0	285	57.2	**2113**	**35.9**
PCT Herlev	70	72.9	70	84.3	107	69.2	297	74.4	353	62.9	337	54.6	**1234**	**65.7**
PCT Herning	166	38.0	151	47.0	176	56.8	181	63.5	208	67.3	191	54.5	**1073**	**55.3**
PCT Himmerland	54	7.4	174	40.2	172	71.5	178	77.0	198	67.7	224	78.1	**1000**	**64.3**
PCT Holbæk	122	13.9	90	45.6	94	64.9	87	70.1	70	54.3	88	50.0	**551**	**47.5**
PCT Horsens	NE	NE	59	64.4	144	82.6	133	93.2	210	75.7	161	56.5	**707**	**75.1**
PCT Hvidovre	NE	NE	4	0.0	138	41.3	242	66.1	171	63.7	156	64.1	**711**	**59.9**
PCT Køge	60	3.3	101	17.8	98	69.4	91	63.7	64	81.3	20	90.0	**434**	**49.8**
PCT Nordsjælland	239	51.0	209	67.5	225	77.3	244	74.6	234	70.1	161	71.4	**1312**	**68.4**
PCT Nykøbing	194	66.5	206	76.7	204	75.0	194	82.5	193	71.0	179	82.1	**1170**	**75.6**
PCT Næstved	200	69.5	222	71.6	182	79.1	220	83.2	257	81.3	203	76.4	**1284**	**77.0**
PCT Odense	271	52.0	429	54.1	533	59.3	568	52.1	591	49.7	459	47.7	**2851**	**52.5**
PCT Randers	182	71.4	189	83.6	246	87.8	251	86.1	261	91.6	209	87.1	**1338**	**85.3**
PCT Rigshospitalet	96	28.1	122	27.0	95	32.6	84	69.0	97	76.3	63	73.0	**557**	**48.3**
PCT Roskilde	146	63.0	155	61.3	211	65.9	215	55.8	213	63.8	200	71.0	**1140**	**63.5**
PCT Silkeborg	169	66.9	190	86.8	179	81.6	174	93.1	136	84.6	104	79.8	**952**	**82.4**
PCT Slagelse	150	71.3	166	77.7	162	83.3	172	87.2	198	81.3	159	72.3	**1007**	**79.1**
PCT Sydvestjysk Sygehus	134	79.1	136	88.2	154	87.7	156	78.8	196	67.9	139	79.9	**915**	**79.6**
PCT Sønderjylland	257	45.5	271	59.4	315	67.9	279	74.2	252	71.0	197	59.4	**1571**	**63.3**
PCT Thy-Mors	167	16.2	164	0.0	169	75.1	191	65.4	148	36.5	121	39.7	**960**	**39.7**
PCT Vejle	267	89.1	230	85.7	215	77.7	217	72.8	264	66.7	195	60.5	**1388**	**75.9**
PCT Vendsyssel	55	0.0	280	10.7	337	21.7	284	38.4	282	42.9	274	20.4	**1512**	**25.7**
PCT Viborg	510	26.7	191	73.8	189	66.7	178	70.2	165	78.8	113	83.2	**1346**	**55.9**
PCT Ålborg	436	31.4	455	28.4	391	28.6	422	43.1	377	35.5	326	47.9	**2407**	**35.3**
PCT Århus	335	35.2	331	41.1	381	44.4	307	58.0	280	54.3	211	56.4	**1845**	**47.3**
Skt. Lukas Hospice	290	11.7	410	17.3	399	20.6	347	20.2	327	30.3	290	34.1	**2063**	**22.1**
Skt. Maria Hospice	110	83.6	93	89.2	142	89.4	160	77.5	158	53.8	144	73.6	**807**	**76.5**
Svanevig Hospice	132	37.9	121	41.3	155	27.1	178	14.0	160	31.3	188	37.2	**934**	**30.7**
Søndergård Hospice	2	0.0	225	20.4	230	47.4	280	81.1	295	65.8	247	60.7	**1279**	**56.8**
**Total**	**6888**	**39.4**	**7928**	**42.9**	**8528**	**51.2**	**8983**	**54.9**	**9095**	**55.2**	**7885**	**52.8**	**49,307**	**49.9**

*RR* Response rate, *NE* Non-existing data because the service did not exist in that year, *PCT* Palliative care team/service in a hospital

Most patients were categorized in the response rate group ≥60.0% but 42.3% were categorized in a lower group, i.e. were admitted to a service having a response rate below 60.0% in the year of admission (Table 1).

### Are symptom scores biased in services with low response rates?

**Fig. 1:**
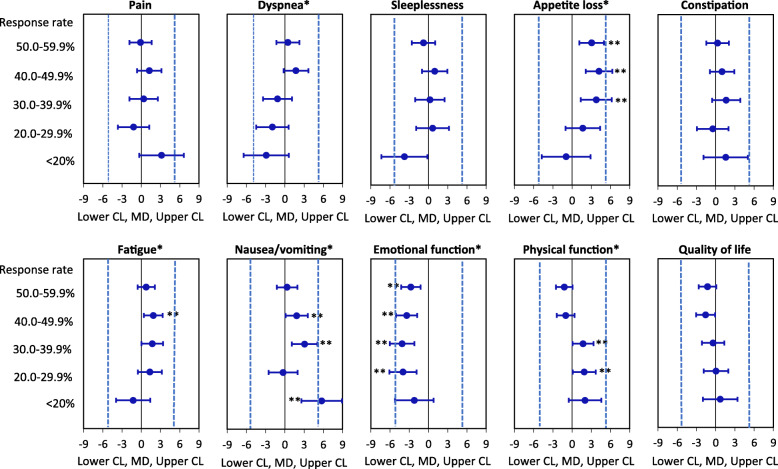
Association between response rate (≥60.0% is reference) and QOL scores from multiple linear regression analyses. Analyses adjusted for type of SPC service (hospice vs. palliative care team) and with random effect of patient id and SPC service. CL = confidence limit, MD = mean difference. For the symptom scales, a negative MD means that the < 60.0% response rate groups have lower symptom scores than the ≥60.0% response rate group. For functional and QOL scales, a positive MD means that the < 60.0% response rate groups have higher functioning/QOL than the ≥60.0% response rate group. Blue dotted lines show the minimum level for clinically relevant mean differences. *Overall *p*-value for the association between response rate and scale score was < 0.05. **P-value for difference in symptom score between the ≥60.0% response rate group and one of the lower response rate groups was < 0.05

**Fig. 2:**
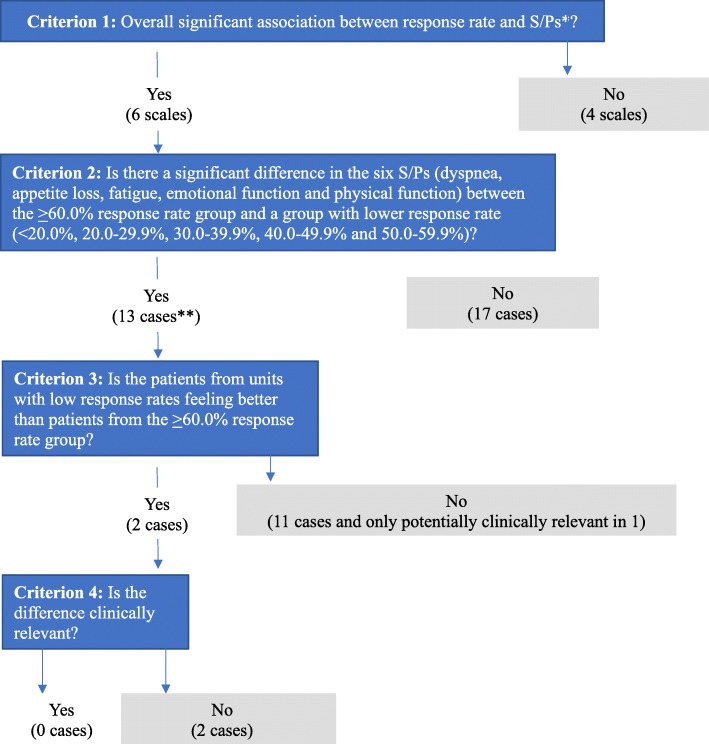
Graph showing how the study hypothesis is tested in the study according to four criteria. *S/Ps = the nine symptoms/problems and overall QOL. **A ‘case’ for each pairwise comparison of S/P mean scores between the ≥60.0% response rate group and a lower response rate group, i.e. five comparisons for each S/P

The results of the multiple linear regressions are shown in Fig. 1 and are summarized in Fig. 2. The results were interpreted using the four criteria listed in the Methods section.

Criterion 1. Significant associations with response rate were found for six of the ten symptoms/problem scales (dyspnea, appetite loss, fatigue, nausea, emotional function and physical function).

Criterion 2. Mean scores for the six symptoms/problems were compared between the ≥60.0% response rate group and each of the five lower response rate groups (< 20.0%, 20.0–29.9%, 30.0–39.9%, 40.0–49.9% and 50.0–59.9%, respectively), i.e., in total 30 comparisons. In 13 of the 30 comparisons, a significant difference was found between the ≥60.0% response rate group and the lower response rate group.

Criterion 3. In two of these 13 comparisons, patients from the lower response rate groups (20.0–29.9% and 30.0–39.0%) had better scores (higher mean physical function) compared to the ≥60.0% response rate group, in accordance with the study hypothesis.

The remaining 11 of the 13 significant comparisons contradicted the study hypothesis as the patients from the lower response rate group had worse symptoms/problems. Thus, compared to the ≥60.0% response rate group, the 30.0–59.9% response rate groups had worse appetite loss, the 40.0–49.9% response rate group had worse fatigue, the < 20.0–49.9% response rate groups had worse nausea and the 20.0–59.9% response rate groups had lower emotional function.

Criterion 4. For physical function, the 20.0–29.9% and 30.0–39.0% response rate groups had 1.9 and 1.7 point higher mean physical function, respectively, compared to the ≥60.0% response rate group and these differences were therefore not clinically relevant. For the 11 significant comparisons that were not in accordance with the study hypothesis, only one was possibly clinically relevant (patients from the < 20.0% response rate group reported 5.7 point more nausea compared to patients from the ≥60.0% response rate group).

### Choice of regression analysis method

The linear regression analyses found more significant associations between response rates and scale scores than the logistic regression analyses did, and thus the linear regression analyses did not generally miss significant associations, except for QOL but in that case the logistic regression analysis did not find systematically worse (or better) QOL in the lower response rate groups compared to the highest response rate group (Table 3).

**Table 3:** *P*-values for the association between response rate and symptom scores in linear and logistic regression

	PA	DY	SL	AP	CO	FA	NV	EF	PF	QOL
Linear	0.11	**0.02**	0.10	**< 0.01**	0.53	**0.04**	**< 0.01**	**< 0.0001**	**< 0.01**	0.19
Logistic	0.96	0.46	0.27	0.10	0.66	**< 0.01**	**0.01**	**< 0.01**	**0.01**	**0.03**

Analyses adjusted type of SPC service (hospice vs. palliative care team) and with random effect of SPC service and patient id

*PA* Pain (*N* = 24.482), *DY* Dyspnea (*N* = 24.255), *SL* Sleeplessness (*N* = 24.256), *AP* Appetite loss (*N* = 24.272), *CO* Constipation (*N* = 24.061), *FA* Fatigue (*N* = 23.698), *NV* Nausea (*N* = 24,291), *EF* Emotional function (*N* = 23,018), *PF* Physical function (*N* = 24,056), *QOL* Quality of life (*N* = 21,043)

Change in mean scale scores when patients from SPC services with response rates < 60.0% are excluded.

**Fig. 3:**
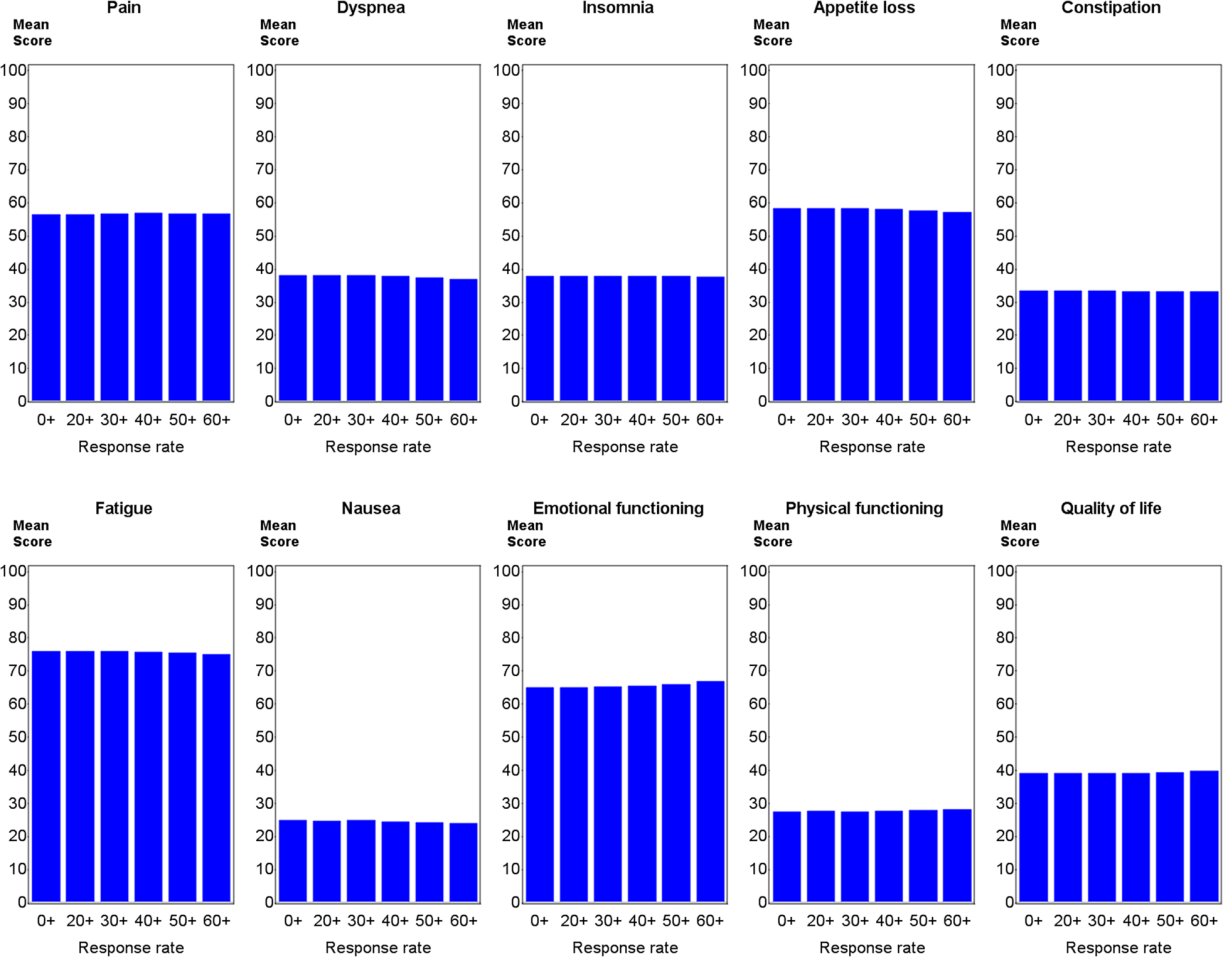
Mean scale scores for SPC services with response rates of ≥0.0%. The whole population (*N* = 24,589), ≥20**.0**% (*N* = 23,987), ≥30**.0**% (*N* = 22,705), ≥40**.0**% (*N* = 20,560), ≥50**.0**% (*N* = 17,634) and ≥ 60.0% (*N* = 14,192), respectively

The mean scale scores were almost identical for the whole population and the sub-populations with exclusion of the < 20.0, < 30.0, < 40.0, < 50.0 and < 60.0% response rate groups, respectively. The largest change in mean score after exclusion of the lower response rate groups was 2.0 (in emotional function, range 0–100) (Fig. 3). Thus, removing patients from services with lower response rates from the mean scale score calculations had almost no effect on the mean scores.

## Discussion

### Evidence supporting selection bias in SPC services with low response rates?

Our aim was to test whether the response rate was associated with scale scores of ten quality of life aspects. By doing this we could test the study hypothesis, i.e. that patients from SPC services with low response rates were reporting better QOL than patients from services with high response rates, indicating possible selection bias in services with low response rates. Such selection bias could happen if the health care professional in low response services had primarily asked the most well patients to answer the EORTC QLQ-C15-PAL questionnaire, leading to an overestimation of the QOL in the low response services which would result in better scores on the ten QOL aspects (lower symptom scores and higher function and overall QOL scores) in these low response services compared to high response services where patients were not selected (or less selected). On the other hand, if patients in low response services were randomly asked to answer the EORTC QLQ-C15-PAL questionnaire the QOL would be representative for the patients in the low response service. This would likely result in similar QOL scores in the low and high response services or perhaps worse scores in the low response services if the explanation to the lower response rate was a sicker patient population. Thus, to accept the study hypothesis, the score of one or more of the quality of life aspects should be significantly better (i.e., lower symptom score, higher function score, higher overall QOL score) in palliative care services with low response rates compared to patients from services with high response rates. However, results from the multiple linear regression analyses did not support the study hypothesis: there were overall associations between response rate and six out of ten QOL scale scores. However, within these six scales, only two out of 30 comparisons were significant and in the expected direction. The magnitude of these comparisons was very small (below 2 on a 0–100 scale) and thus not clinically relevant. Furthermore, mean scale scores in the whole population were almost identical to mean scale scores calculated after exclusion of the < 20.0, < 30.0, < 40.0, < 50.0 and < 60.0% response rate groups, respectively, supporting that the response rate had neglectable impact on the scale scores. This is in accordance with findings from a previous, much smaller study in patients with advanced cancer, where no evidence of clinically relevant difference in the quality of life scores due to non-participation was found [6].

There were some cases where patients from services with low response rates reported significantly worse QOL, and not better QOL as hypothesized. The differences were relatively small (1.8–5.7 on a 100-point scale) and due to the large number of statistical test performed, these findings may be due to chance. It is however not unlikely that non-response is associated with poor health [4, 5] and the slightly worse symptom/problem scores in SPC services with low response rates may reflect a slightly more ill patient population in SPC services with low response rates, i.e. not selection bias.

### Strengths and weaknesses

There are several strengths in this study. First, it is based on a large dataset with national coverage including data from all SPC services in Denmark. Second, the possibility that different SPC services may not have the same composition of patients (due to socio-demographic or other differences across referral areas) was accounted for by including the random effect of specific SPC service.

No services had complete data, which is a limitation for at least two reasons. First, because our conclusion that there is not more selection bias in SPC services with low response rates than in those with ≥60.0% response rate is not a full investigation of the possibility of selection bias in SPC services with low response rates. However, due to the nature of SPC, which is provided to patients with very advanced disease and short survival, it will probably not be possible to achieve much higher response rates than achieved in the best performing services. Second, it is impossible to know whether differences in scale scores between SPC services with high and low response rates are in fact do to selection bias in SPC services with low response rates or whether they are caused by SPC services with low response rates actually having different scale scores, since we do not know the true scale scores. Thus, looking at difference in scale scores between SPC services with low and high response rates is only one way to attempt to decide whether selection bias seems plausible in services with low response rates, and the fact that we found no major impact is reassuring.

### Future research

Only a few large studies (1000–2000 patients) have looked at symptom prevalence or symptom levels in patients at the start of palliative care [17, 20, 21] and to our knowledge this is the first European study to report data from all services in a whole country. Given that the present study indicates that our full data set can be used for analysis without any major risk of impact of selection bias, future analyses can safely be made at the national level to investigate the symptoms/problems and QOL among patients admitted to SPC.

## Conclusion

The study hypothesis – that patients from SPC services with lower response rates have better scores on different QOL aspects than those from SPC services with high response rates due to selection bias in services with low response rates - was not supported. Therefore, there does not seem to be any reason to exclude data from SPC services with low response rates in future studies of national data sets.

## Data Availability

The data utilized in this study are available at Danish Palliative Care Database. Restrictions apply to the availability of these data.

## Abbreviations

DPD: Danish Palliative Care Database
SPC: Specialized palliative care
QOL: Quality of life
